# Possible ramifications of climate variability on HPAI-H5N1 outbreak occurrence: Case study from the Menoufia, Egypt

**DOI:** 10.1371/journal.pone.0240442

**Published:** 2020-10-29

**Authors:** Yumna Elsobky, Gamal El Afandi, Ehsan Abdalla, Ahmed Byomi, Gopal Reddy

**Affiliations:** 1 Department of Hygiene and Zoonosis, Faculty of Vet. Medicine, University of Sadat City, Sadat City, Egypt; 2 College of Agriculture, Environment and Nutrition Sciences, Tuskegee University, Tuskegee, Alabama, United States of America; 3 Astronomy and Meteorology Department, Faculty of Science, Al-Azhar University, Cairo, Egypt; 4 Department of Graduate Public Health, College of Veterinary Medicine, Tuskegee University, Tuskegee, Alabama, United States of America; 5 Pathobiology Department, College of Veterinary Medicine, Tuskegee University, Tuskegee, Alabama, United States of America; Faculty of Science, Ain Shams University (ASU), EGYPT

## Abstract

Long endemicity of the Highly Pathogenic Avian Influenza (HPAI) H5N1 subtype in Egypt poses a lot of threats to public health. Contrary to what is previously known, outbreaks have been circulated continuously in the poultry sectors all year round without seasonality. These changes call the need for epidemiological studies to prove or deny the influence of climate variability on outbreak occurrence, which is the aim of this study. This work proposes a modern approach to examine the degree to which the HPAI-H5N1disease event is being influenced by climate variability as a potential risk factor using generalized estimating equations (GEEs). GEE model revealed that the effect of climate variability differs according to the timing of the outbreak occurrence. Temperature and relative humidity could have both positive and negative effects on disease events. During the cold seasons especially in the first quarter, higher minimum temperatures, consistently show higher risks of disease occurrence, because this condition stimulates viral activity, while lower minimum temperatures support virus survival in the other quarters of the year with the highest negative effect in the third quarter. On the other hand, relative humidity negatively affects the outbreak in the first quarter of the year as the humid weather does not support viral circulation, while the highest positive effect was found in the second quarter during which low humidity favors the disease event.

## Introduction

Avian influenza posed a significant pandemic threat [1, 2] through the persistence of mutant or genetically reassorted progenitor in poultry [3]. HPAI outbreaks have been clustered globally in three Asian countries -Bangladesh, Indonesia and Vietnam and in Middle east in Egypt [4] with enzootic H5N1-HPAI clinical disease in poultry [5]. Outside Asia the longest endemic status of H5N1-HPAI is reported in Egypt [6]. The highest human cases were recorded in Egypt with a case fatality rate coming to 53.2% according to the World Health Organization (WHO) [7]. A lot of studies evidenced that H5N1 increase its adaptation in human through antigenic drift, considering Egypt a hotspot for the evolution of a pandemic potential virus [8, 9]. Disease eradication in poultry is crucial [3] since reported human case locations overlap significantly with reported A (H5N1) in poultry outbreaks areas [10].

The spread of the virus is investigated by many studies with a discussion of the possible risk factors. However, the climate factor, which can impact the outbreak, has been enormously overlooked [11]. The global trend of HPAI-H5N1 influenza outbreak shows a clear seasonality [3] with the seasonal peak occurrence, particularly, in countries with endemic H5N1-HPAI clinical disease in poultry [4, 12, 13]. There is a lot of evidence proved that the avian influenza outbreak is related to climate change [14, 15]. Climate variability changes the ability of the virus to survive and it also could be a possible factor in the widening of H5N1 infection if it causes longer environmental survival [10, 16–19]. So, it’s considered one of the driving forces of the outbreaks [15, 20].

Countries like Egypt facing significant changes in their climate likely experience a higher risk of outbreaks [15].Understanding climate variability factors may enable the expectation and control of future disease events in Egypt [6]. Unfortunately, the environmental persistence of A/H5N1 is troublesome to evaluate [21] and prediction based on climate variability has once in a while been assessed, to fill the information crevice, the evidence is required for the effect of them on HPAI-H5N1 outbreaks [22, 23]. The study proposes a new approach to investigate the degree to which the HPAI-H5N1event is being influenced by climate variability as a potential risk factor in the spread and maintenance of the virus in the environment using generalized estimating equations (GEEs).

## Data and methods

**Fig 1:**
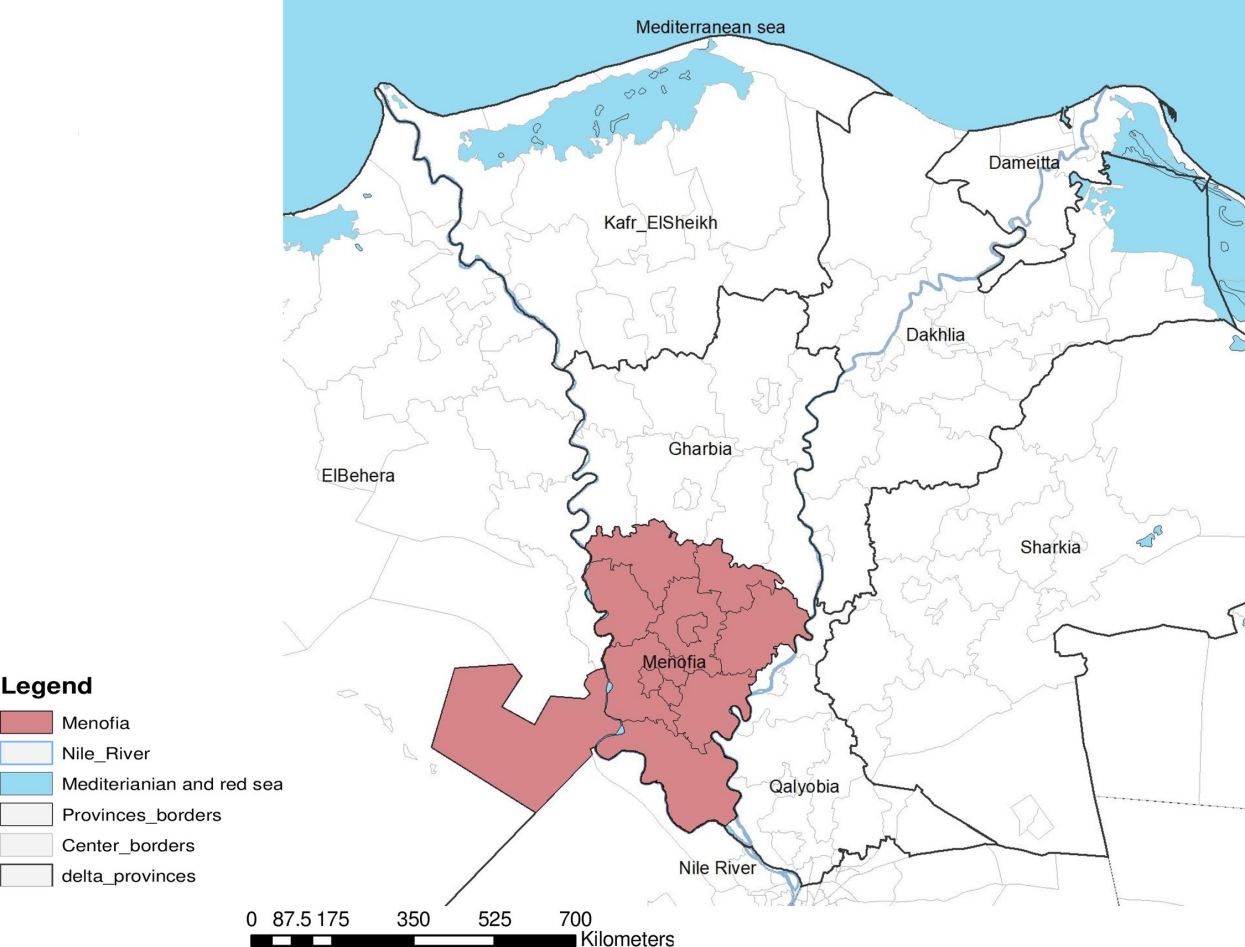
Menoufia governorate, Nile Delta, Egypt.

This study has been carried out using the extent of one of the Nile Delta governorates (Menoufia, Egypt) as highlighted in (Fig 1), reported as one of the highest disease incidence [24]. In addition to that Menoufia is considered one of the leading poultry producing governorates in Egypt [25].

An outbreak is the unit of analysis, based on that all poultry populations in each single village were considered infected with HPAI-H5N1even if there was only one reported outbreak within a certain circumscribed location in this village at a certain point in time. The outbreak is defined as the smallest epidemiological unit, it may represent farms, household or whole villages. Each case was Geo-referenced by GPS coordinates where HPAI-H5N1had been identified at a definite time. The country map was constructed by ArcGIS 10.5 software (Environmental Systems Research Institute, Inc., Redlands, CA, USA) (Fig 1).

Domestic poultry HPAI-H5N1 outbreak data have been collected from the Egyptian ministry of agriculture (Egyptian Committee for Veterinary Services) official reports for national surveillance. In addition to the database of the Global Animal Health Information System of the Food and Agricultural Organization (FAO) were obtained from the Emergency Prevention System for Transboundary Animal and Plant Pests and Diseases Programme EMPRES-I [26]. All are integrated into one dataset for the study period from January 2006 to December 2016.

Climatological data from January 2006 to December 2016 were obtained from the NASA Prediction of Worldwide Energy Resources [27]. Parameters are based on solar radiation delivered from satellite observation and meteorological data from assimilation models. Climate parameters for the outbreaks in each Epidemic Wave (EW) from EW1 to EW6 were extracted based on their spatial coordinates and outbreak dates to establish its spatial linkage with HPAI-H5N1 outbreaks. The internal environment as temperature and relative humidity is typically near to outside conditions in naturally ventilated houses [28, 29]. The outbreaks can, therefore, be predicted based on external conditions. The meteorological parameters used in this study, units and definition, are shown in Table 1.

**Table 1:** Meteorological parameters units and definition.

PARAMETER	UNITS	DEFINITION
**RELATIVE HUMIDITY (RH)**	**%**	Ratio of actual partial press of water vapor to the partial pressure at saturation, expressed in percent. The daily average of relative humidity at 2 meters above the surface of the earth.
**DEW/FROST POINT (T-DEW)**	**˚C**	Temperature at which air is saturated with water vapor. The daily average of dew/frost point temperature is at 2 meters above the surface of the earth and calculated as the average of hourly values pulled from the MERRA-2 assimilation model.
**MAXIMUM TEMPERATURE (T-MAX)**	**˚C**	The daily maximum temperature at 2 meters above the surface of the earth.
**MINIMUM TEMPERATURE (T-MIN)**	**˚C**	The daily minimum temperature at 2 meters above the surface of the earth.
**MAXIMUM WIND SPEED (WS-MAX)**	**m/s**	The daily Maximum wind speed at 2 meters above the surface of the earth.
**MINIMUM WIND SPEED (WS-MIN)**	**m/s**	The daily Minimum wind speed at 2 meters above the surface of the earth.

### Design, statistical analysis and modelling

**Fig 2:**
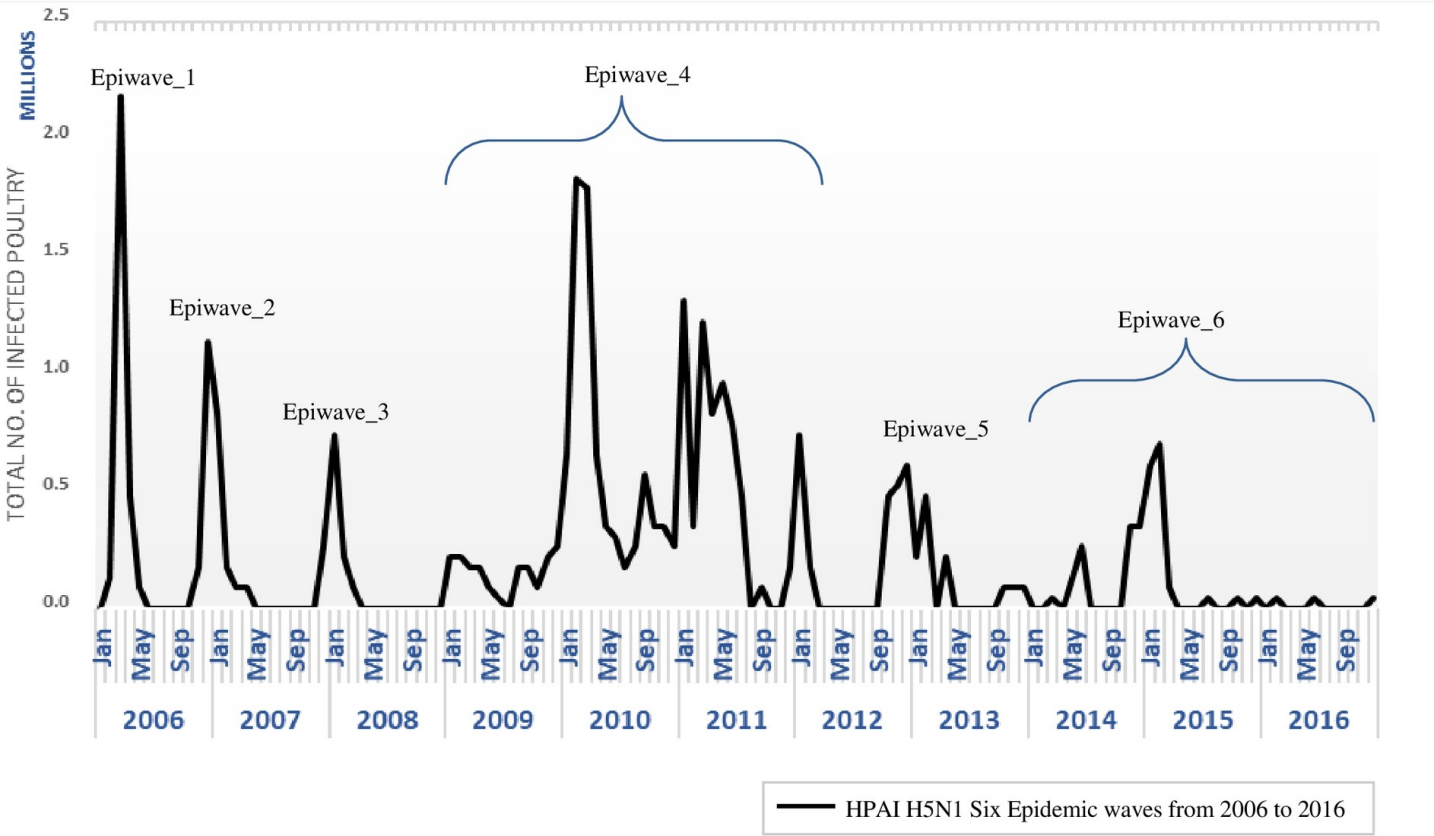
Epidemic curve.

To display the overall dynamics of HPAI-H5N1 outbreaks, a time series was constructed to display temporal distribution using reported outbreaks from 2006 to 2016 at Menoufia, Egypt. This study based mainly on the Epidemic waves of the disease in the study area as illustrated in (Fig 2).

In order to test if climate variability has any relation with outbreak occurrence, General Linear Model (GLM) has been used to test the difference between climatic conditions associated with outbreak periods (Epidemic waves) and those with outbreak free periods. Then, we investigated the existence of differences in climate parameters that accompanied the outbreaks and whether these differences were seasonally or monthly or even in four Quarters of the year, GLM is chosen to test these differences.

Finally, based on the results from GLM, Poisson regression using generalized estimating equations (GEEs) is done to investigate the degree to which the HPAI-H5N1 event is being influenced by climate variability as a potential risk factor in the spread and maintenance of the virus in the environment. Model is based on epidemic waves outbreak data with repeated measurements from the four quarters of the year over the period 2006–2016 from Menofia, Egypt. This study used a GEE model corrected through an offset parameter for the poultry population sizes. It also accounted for over-dispersion for the number of poultry outbreaks, because one outbreak can result in other outbreaks via local transmission.

Estimated coefficients antilog (bi) corresponds to the relative risk (RR). Univariate analysis for each variable was conducted, and those with a significance level p < 0.010 were considered in the results. SAS version 9.4 was used for all statistical analyses.

## Results and discussion

**Fig 3:**
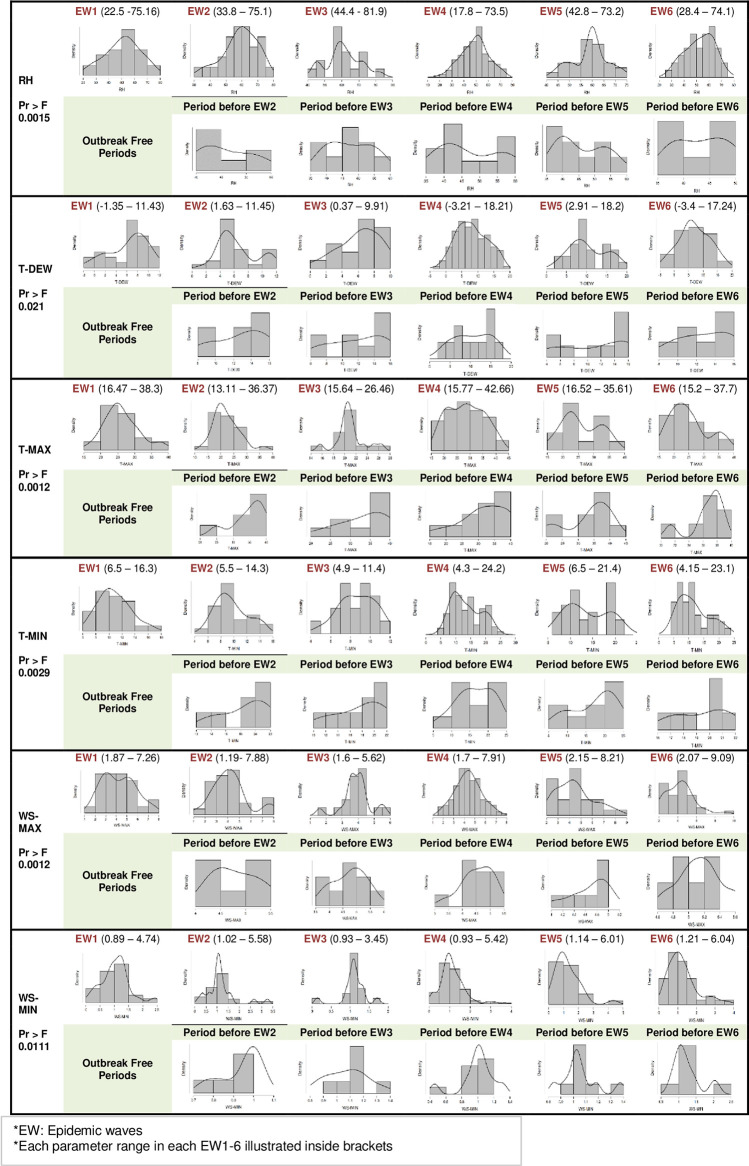
Line graphs indicate differences in climate trend between HPAI H5N1 outbreak (EWs) and the outbreak free periods tested by General Linear Model (GLM).

In Egypt, HPAI-H5N1 has been reported for the first time in poultry in 2006 [6]. From 2006 to the end of 2016, six epidemic waves of HPAI-H5N1 of about 700 outbreaks were observed in the Menoufia governorate as illustrated in (Fig 2). During epidemic waves, the number of outbreaks increases to a peak then falls until the wave is over. (Fig 2) indicates the beginning, peaking and ending period of outbreaks over six epidemic waves. Through visual inspection of (Fig 3), it might be possible to give the different climate parameters distribution in outbreak periods (Epidemic waves) the same description of epidemic wave collectively from the way it begins, peaks then end, unlike the distribution of the same parameters in outbreak free periods.

During 2006–2008, HPAI-H5N1 infection is associated with winter months in Egypt [30, 31]. When the environmental temperature increased in summer and autumn seasons, the infection decreased. The pattern of outbreaks in first, second and third epidemic waves (EW1, EW2, and EW3) from 2006 to 2008 were observed to have one definitive peak for each wave, (Fig 2). It seems to be in harmony with the pattern of different climate parameters, but it is not perfectly identical as one can observe in the distribution curves in (Fig 3).

However, by 2009, the virus has been circulated and reported all over the year [30, 31]. The fourth epidemic wave (EW4) appears to have several successive peaks, also it lasts for a longer time in comparison to other precede waves. This could be attributed to newly emerged clades thermostability (2.2.1.1 and 2.2.1.2-B/2.2.1.2-C), increase virus ability to survive at elevated temperatures (56 ^0^C degrees) [6]. These clades lead to maintain endemicity of HPAI-H5N1 through virus adaptation in the environment in Egypt [24].

The distribution of climate parameters during EW4 surprisingly follows the same pattern of the outbreak, especially in T-MAX and T-MIN. The outbreak in the last two waves (EW5, EW6) appears to occur also with several peaks but the epidemic curve does not upsurge to reach high levels as EW4, the way it became more flattened but still posse peaks that clearly can be observed, (Fig 2). T-MAX, T-MIN and WS-MAX curves in last two epi-waves appear to follow the same pattern becoming more flattened in comparable to the same parameter curve in other epidemic waves, (Fig 3).

Besides, it could be evidenced from GLM results and the distribution curves in (Fig 3) that the climate trend significantly differ between HPAI-H5N1 outbreak periods (EWs) and outbreak free periods. Therefore, climate conditions and its variability might be considered as one of the driving forces of the outbreaks as shown by [15, 20]. In addition, it may influence the outbreak occurrence by altering the virus’s ability to survive in the environment and it also could be a possible risk factor in the widening and spread of HPAI-H5N1 infection [17–19].

Meanwhile, the outbreak might occur nearly under the same climatic conditions. It is indicated by comparing the climate conditions and their variability across different epidemic waves in (Fig 3). Although the outbreaks were widely distributed and it prompts the range of climate parameters to be conceivably wide [11], there are ranges in common in all epidemic waves could be used to determine highest risk range for outbreaks occurrence, (44.4–73.2), (16.52–26.46), (6.5–11.4), (2.15–5.62) for RH, T-MAX, T-MIN, WS-MAX respectively.

**Fig 4:**
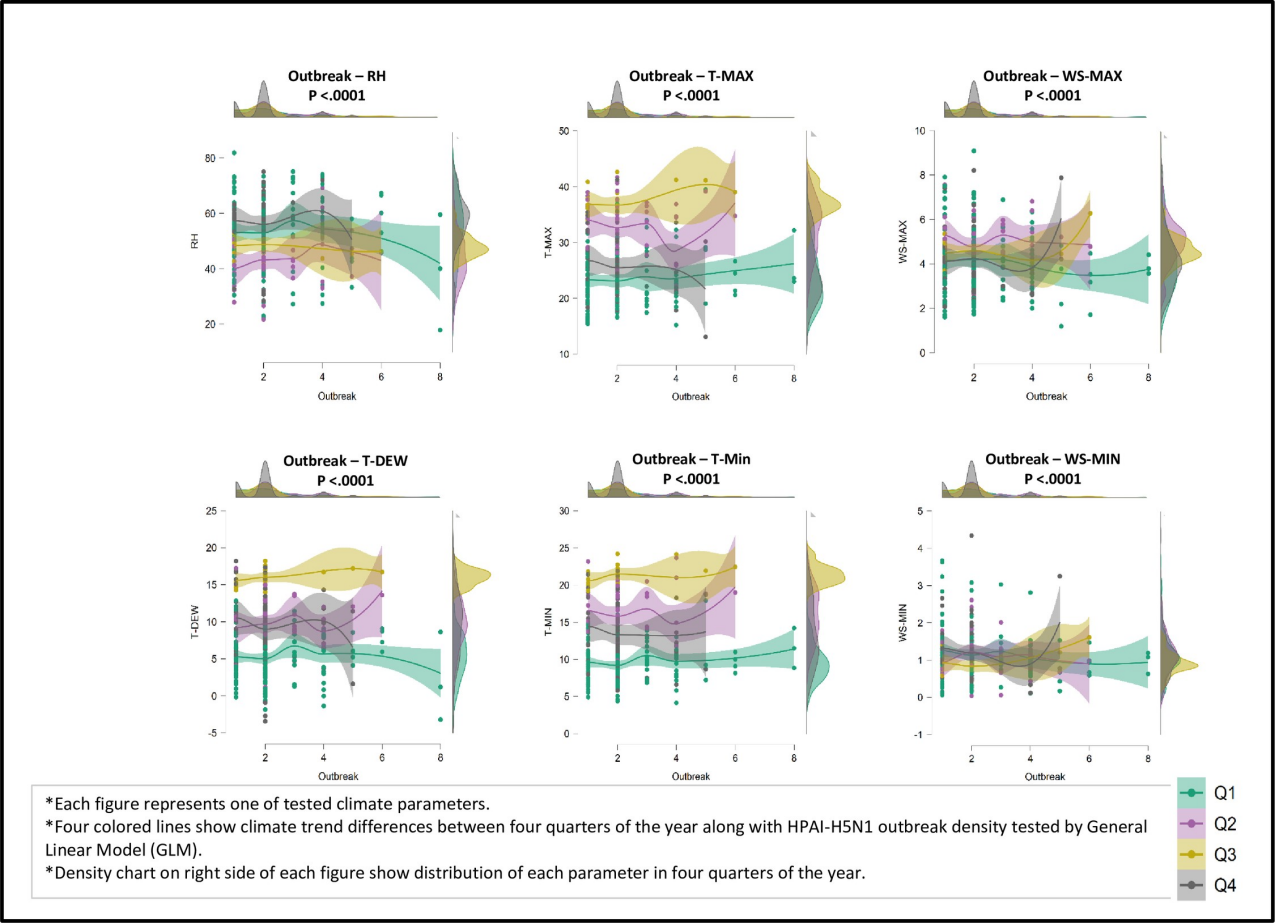
Climate trend differences between four quarters of the year along with HPAI-H5N1 outbreak density tested by General Linear Model (GLM).

The climatic parameters with seasonally and monthly outbreak occurrence found to be not significantly different. But climate trend differs significantly between four quarters of the year along with HPAI-H5N1 outbreak density tested by General Linear Model (GLM), results in (Fig 4). The infection was basically concentrated from October to March, these results are similar to the finding by [12] and in accordance to Egyptian surveillance [6, 32–36]. In addition, many other scientists declared the same observations globally [14].

Despite the seasonality nature of Avian Influenza, without adequate and more effective mitigation strategies horizontal span of the epidemic curve would extend for longer periods without predictable seasonality. Increasing the duration of epidemic waves could be accompanied by continuing outbreaks extend sometimes to the whole year encountered in severe economic losses. Control measures have been applied appear to have a short-term impact in effectively lessening the disease events and are not exceptionally effective in compressing the disease spatially and change the regularity of the H5N1 epidemic [2, 11].

**Fig 5:**
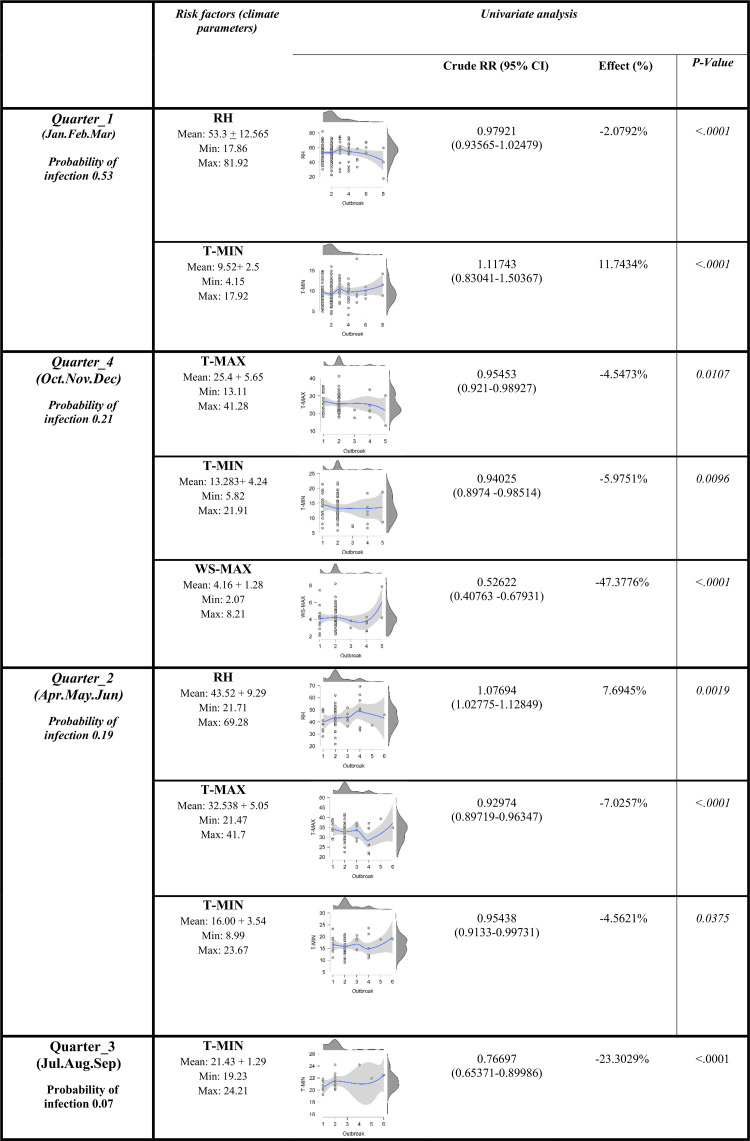
The association between the number of HPAI-H5N1 outbreaks in poultry and climate parameters as a risk factor^a^. ^a^The result for the number of poultry outbreaks was obtained by Poisson regression using generalized estimating equations (GEEs) based on epidemic waves outbreak data with repeated measurements from the four quarters of the year (ordered from the highest probability of infection to the lowest) over the period 2006–2016 from Menofia, Egypt.

According to the previous results, only significant parameters are included in the generalized estimating equations model (GEEs) and the model was developed based on four quarters of the year considering all epidemic waves from 2006–2016 as presented in (Fig 5). The highest probability of infection is observed in the first quarter of the year, followed by the fourth and the second one respectively as shown in (Fig 5). While the lowest one was recorded in the third quarter. These results are in accordance with the incidence of outbreaks in previous surveillance in Egypt peaked from October 1^st^ to March 31^st^ of each year [6, 32–36], hence the first and fourth quarters of the year.

Results from the GEEs model indicate that not all parameters have the same effect on the outbreak over all periods. All possible effects of climate variability on disease events in four quarters of the year will be discussed as given in (Fig 5), considering only significant parameters.

**In the first quarter**, the outbreak is significantly associated with minimum temperature and relative humidity (p <0.01), while the rest of the climatic parameters show no significant effect on the outbreak occurrence. It is conducted from the minimum temperature positive estimated coefficient that outbreak likelihood increases as minimum temperatures rise. One can say that a one-unit minimum temperature increase, lead to an outbreak risk rise to about 12%. While for relative humidity; one-unit increase, led to lowering the outbreaks to 2%.

**In the fourth quarter**, the outbreak is significantly associated with the minimum, maximum temperature and maximum wind speed (p <0.01), while the rest of the climatic parameters show no significant effect on the outbreak occurrence in the model. The parameters estimated coefficient is all negative, proposing that with their rise, the outbreak likelihood diminishes. An increase of one-unit minimum and maximum temperatures decreases the outbreak risk by 6%, and 5% respectively. While a one-unit increase in maximum wind speed; lowered the outbreaks to 47%.

**In the second quarter**, the outbreak is significantly associated with the minimum, maximum temperatures and relative humidity (p <0.01), while the rest of the climatic parameters show no significant effect on the outbreak occurrence in the model in the second quarter. Relative humidity positive estimated coefficient suggesting that the probability of outbreak and relative humidity rise together. A one-unit relative humidity increase raised the outbreak by about 8%. While a one-unit increase in both minimum and maximum temperatures; lowered the outbreaks by 5% and 7% respectively.

**In the third quarter**, the outbreak is significantly associated only with minimum temperature (p <0.01), while the rest of the climatic parameters show no significant effect on the outbreak occurrence in the model. The estimated coefficient is negative proposing that outbreak likelihood diminishes as minimum temperature rises. A one-unit increase in minimum temperature could increase the outbreak risk by about 23%.

Higher minimum temperature in the first quarter months would increase the disease likelihood while lower humidity would likely increase the likelihood of the disease. These results previously observed by different studies [15, 22, 23, 37, 38]. A Higher risk of disease in the cold seasons was observed during higher minimum temperatures as the warm temperature in such a condition is considered a significant risk factor because it can stimulate the activity of the virus. This finding is in good agreement with different scientists [18, 22, 38, 39].

Other scientists show that lower temperatures increase virus stability by supporting better survival of the virus in the environment [15, 40–42]. This could contribute to the negative association of an outbreak occurrence with the minimum and maximum temperature in the other quarters of the year. [15] showed that outbreaks likelihood decline by 0.22% for a1-degree Celsius rise in temperature.

One can find that the highest negative effect of minimum temperature was found in the third quarter (April, May, and June), which can decrease the outbreak by 23% for a one-unit increase, the same finding observed in Egypt by [6].

In addition to that exceptionally high humidity could decrease the likelihood of HPAI-H5N1 since during such condition humid weather do not support virus survival and disease occurrence, which is also supported by [37]. In other circumstances, low humidity months represent favorable conditions to the outbreak occurrence [37]. This could explain why relative humidity negatively affects the outbreak in the first quarter, while the highest positive effect of relative humidity was found in the second quarter months (April, May, and June), that can increase the outbreak by 8% for a one-unit increase.

Eventually, climatic factors alone couldn’t explain HPAI-H5N1 prevalence in poultry [6], rather than combination of key risk variables that might give a reasonable explanation [37, 43].

## Summary and conclusion

Climate variability has been proved to influence the outbreak occurrence and spread of the virus in the environment. The study infers that the HPAI-H5N1 outbreak at different epidemic waves occurs nearly in the same conditions. From which, an expected risk range of each climatic parameter for HPAI-H5N1 disease event could be obtained. Despite the nature of AI is seasonality, outbreaks will continue to occur all over the year with observed thermostability of HPAI-H5N1 newly emerged clades.

The effect of climate variability differs according to the timing of outbreak occurrence. Temperature and relative humidity could have both positive and negative effects of disease events. During the cold seasons especially in the first quarter, higher minimum temperatures, consistently show higher risks of disease occurrence, because this condition stimulates viral activity, while lower temperature support virus survival in the other quarters of the year with the highest effect in the third quarter. When humidity is exceptionally high (close to 100%), it is noticed that the disease events decrease since the humid weather does not support virus survival in the environment, which is observed in the first quarter. In other circumstances, low humidity favors the occurrence of the disease as in the second quarter with highest positive effect.

## Recommendation

Using climate parameter could help to enhance HPAI-H5N1 targeted surveillance program to outbreaks high risk periods. Regular active surveillance along with climate variability should be continued throughout the year to monitor the changes in virus ecology. Therefore, using climate conditions and its variability in mitigation strategies would have co-benefits in diminishing future HPAI-H5N1 disease event.

## Supporting information

S1 Data (XLSX)

## References

[1] Doherty PC, Turner SJ, Webby RG, Thomas PG. Influenza and the challenge for immunology. Nature immunology. 2006;7(5):449 10.1038/ni1343 16622432

[2] Webster RG, Govorkova EA. H5N1 influenza—continuing evolution and spread. New England Journal of Medicine. 2006;355(21):2174–7. 10.1056/NEJMp068205 17124014

[3] Biswas PK, Islam MZ, Debnath NC, Yamage M. Modeling and roles of meteorological factors in outbreaks of highly pathogenic avian influenza H5N1. PloS one. 2014;9(6):e98471 10.1371/journal.pone.0098471 24886857 PMC4041756

[4] Zhang Z, Chen D, Chen Y, Davies TM, Vaillancourt J-P, Liu W. Risk signals of an influenza pandemic caused by highly pathogenic avian influenza subtype H5N1: spatio-temporal perspectives. The veterinary journal. 2012;192(3):417–21. 10.1016/j.tvjl.2011.08.012 21944318

[5] Swayne D, Pavade G, Hamilton K, Vallat B, Miyagishima K. Assessment of national strategies for control of high-pathogenicity avian influenza and low-pathogenicity notifiable avian influenza in poultry, with emphasis on vaccines and vaccination. Revue Scientifique et Technique-OIE. 2011;30(3):839 10.20506/rst.30.3.2081 22435196

[6] Salaheldin AH, Kasbohm E, El-Naggar H, Ulrich R, Scheibner D, Gischke M, et al Potential biological and climatic factors that influence the incidence and persistence of highly pathogenic H5N1 avian influenza virus in Egypt. Frontiers in microbiology. 2018;9:528 10.3389/fmicb.2018.00528 29636730 PMC5880882

[7] Abdelwhab E, Hassan M, Abdel-Moneim A, Naguib M, Mostafa A, Hussein I, et al Introduction and enzootic of A/H5N1 in Egypt: Virus evolution, pathogenicity and vaccine efficacy ten years on. Infection, Genetics and Evolution. 2016;40:80–90. 10.1016/j.meegid.2016.02.023 26917362

[8] Neumann G, Macken CA, Karasin AI, Fouchier RA, Kawaoka Y. Egyptian H5N1 influenza viruses—cause for concern? PLoS pathogens. 2012;8(11):e1002932 10.1371/journal.ppat.1002932 23166487 PMC3499568

[9] Watanabe Y, Ibrahim MS, Ellakany HF, Kawashita N, Mizuike R, Hiramatsu H, et al Acquisition of human-type receptor binding specificity by new H5N1 influenza virus sublineages during their emergence in birds in Egypt. PLoS pathogens. 2011;7(5):e1002068 10.1371/journal.ppat.1002068 21637809 PMC3102706

[10] European Centre for Disease Prevention and Control. Human infection with avian influenza A(H5N1) virus, Egypt–first update. 13 March 2015. Stockholm: ECDC; 2015 2015 10.2807/esw.12.04.03125-en

[11] Zhang Z, Chen D, Liu W, Wang L. Evaluating the Impact of Climate Change on Global HPAI H5N1 outbreaks. International Congress on Environmental Modelling and Software. 2010;36 https://scholarsarchive.byu.edu/iemssconference/2010/all/36/.

[12] Si Y, Skidmore AK, Wang T, De Boer WF, Debba P, Toxopeus AG, et al Spatio-temporal dynamics of global H5N1 outbreaks match bird migration patterns. Geospatial health. 2009:65–78. 10.4081/gh.2009.211 19908191

[13] Loth L, Gilbert M, Osmani MG, Kalam AM, Xiao X. Risk factors and clusters of highly pathogenic avian influenza H5N1 outbreaks in Bangladesh. Preventive veterinary medicine. 2010;96(1–2):104–13. 10.1016/j.prevetmed.2010.05.013 20554337 PMC4874198

[14] Zhao X-Y, Guo S-M, Ghosh M, Li X-Z. Stability and persistence of an avian influenza epidemic model with impacts of climate change. Discrete Dynamics in Nature and Society. 2016;2016 10.1155/2016/7871251

[15] Mu JH, McCarl BA, Wu X, Gan L. Climate Change Influences on the Risk of Avian Influenza Outbreaks and Associated Economic Loss. 2011.

[16] World Health Organization. Influenza at the human-animal interface, Summary and assessment as of 3 March 2015 [11 March 2015]. 2015. http://www.who.int/influenza/human_animal_interface/Influenza_Summary_IRA_HA_interface_3_March_2015.pdf?ua = 1.

[17] Morin CW, Stoner-Duncan B, Winker K, Scotch M, Hess JJ, Meschke JS, et al Avian influenza virus ecology and evolution through a climatic lens. Environment international. 2018;119:241–9. 10.1016/j.envint.2018.06.018 29980049

[18] Gilbert M, Xiao X, Pfeiffer DU, Epprecht M, Boles S, Czarnecki C, et al Mapping H5N1 highly pathogenic avian influenza risk in Southeast Asia. Proceedings of the National Academy of Sciences. 2008;105(12):4769–74. 10.1073/pnas.0710581105 18362346 PMC2290786

[19] Gilbert M, Slingenbergh J, Xiao X. Climate change and avian influenza. Revue scientifique et technique (International Office of Epizootics). 2008;27(2):459 10.20506/rst.27.2.1821 18819672 PMC2709837

[20] Mu JE, McCarl BA, Wu X, Ward MP. Climate change and the risk of highly pathogenic avian influenza outbreaks in birds. International Journal of Environment and Climate Change. 2014:166–85. 10.9734/bjecc/2014/8888

[21] Delquigny T, Edan M, Nguyen D, Pham T, Gautier P. Impact of Avian Influenza Epidemic and Description of the Avian Production in Vietnam. 2004. Food and Agriculture Organization of The United Nations. 2004 10.18356/877ef4ac-en

[22] Si Y, Wang T, Skidmore AK, de Boer WF, Li L, Prins HH. Environmental factors influencing the spread of the highly pathogenic avian influenza H5N1 virus in wild birds in Europe. Ecology and Society. 2010;15(3):26–. 10.5751/es-03622-150326

[23] Liu C-M, Lin S-H, Chen Y-C, Lin KC-M, Wu T-SJ, King C-C. Temperature drops and the onset of severe avian influenza A H5N1 virus outbreaks. PLoS one. 2007;2(2):e191 10.1371/journal.pone.0000191 17297505 PMC1794318

[24] Arafa A, El-Masry I, Kholosy S, Hassan MK, Dauphin G, Lubroth J, et al Phylodynamics of avian influenza clade 2.2. 1 H5N1 viruses in Egypt. Virology journal. 2016;13(1):49 10.1186/s12985-016-0477-7 27000533 PMC4802640

[25] ElMasry I, Elshiekh H, Abdlenabi A, Saad A, Arafa A, Fasina FO, et al Avian influenza H5N1 surveillance and its dynamics in poultry in live bird markets, Egypt. Transboundary and emerging diseases. 2017;64(3):805–14. 10.1111/tbed.12440 26608470

[26] Food and Agriculture Organization. EMPRES‐i‐Global Animal Disease Information System.2016http://empres-i.fao.org/eipws3g/#h = 0

[27] National Aeronautics and Space Administration. Prediction of Worldwide Energy Resources. POWER Release-8; Retrived from https://power.larc.nasa.gov/#resources. 2019.

[28] Chepete H, Tsheko R. Hot and cold weather heat load dynamics of uninsulated broiler house in Botswana. Agricultural Engineering International: CIGR Journal. 2006 10.13031/2013.18408

[29] Czarick M III, Fairchild B, Daghir N. Poultry housing for hot climates. Poultry production in hot climates. 2008;80 10.1079/9781845932589.0080

[30] Aly M, Arafa A, Hassan M. Epidemiological findings of outbreaks of disease caused by highly pathogenic H5N1 avian influenza virus in poultry in Egypt during 2006. Avian diseases. 2008;52(2):269–77. 10.1637/8166-103007-Reg.1 18646456

[31] Hafez M, Arafa A, Abdelwhab E, Selim A, Khoulosy S, Hassan M, et al Avian influenza H5N1 virus infections in vaccinated commercial and backyard poultry in Egypt. Poultry science. 2010;89(8):1609–13. 10.3382/ps.2010-00708 20634514

[32] Arafa A, Naguib M, Luttermann C, Selim A, Kilany W, Hagag N, et al Emergence of a novel cluster of influenza A (H5N1) virus clade 2.2. 1.2 with putative human health impact in Egypt, 2014/15. Eurosurveillance. 2015;20(13):21085 10.2807/1560-7917.es2015.20.13.21085 25860390

[33] Arafa A, Suarez D, Kholosy S, Hassan M, Nasef S, Selim A, et al Evolution of highly pathogenic avian influenza H5N1 viruses in Egypt indicating progressive adaptation. Archives of virology. 2012;157(10):1931–47. 10.1007/s00705-012-1385-9 22760662

[34] Arafa A-S, Hagag NM, Yehia N, Zanaty AM, Naguib MM, Nasef SA. Effect of cocirculation of highly pathogenic avian influenza H5N1 subtype with low pathogenic H9N2 subtype on the spread of infections. Avian diseases. 2012;56(4s1):849–57. 10.1637/10152-040812-Reg.1 23402103

[35] El-Zoghby EF, Aly MM, Nasef SA, Hassan MK, Arafa A-S, Selim AA, et al Surveillance on A/H5N1 virus in domestic poultry and wild birds in Egypt. Virology journal. 2013;10(1):203 10.1186/1743-422X-10-203 23799999 PMC3699397

[36] Kayali G, Kandeil A, El-Shesheny R, Kayed AS, Maatouq AM, Cai Z, et al Avian influenza A (H5N1) virus in Egypt. Emerging infectious diseases. 2016;22(3):379 10.3201/eid2203.150593 26886164 PMC4766899

[37] Tran C, Yost R, Yanagida J, Saksena S, Fox J, Sultana N. Spatio-temporal occurrence modeling of highly pathogenic avian influenza subtype H5N1: A case study in the Red River Delta, Vietnam. ISPRS International Journal of Geo-Information. 2013;2(4):1106–21. 10.3390/ijgi2041106

[38] Zhang Z, Chen D, Chen Y, Wang B, Hu Y, Gao J, et al Evaluating the impact of environmental temperature on global highly pathogenic avian influenza (HPAI) H5N1 outbreaks in domestic poultry. International journal of environmental research and public health. 2014;11(6):6388–99. 10.3390/ijerph110606388 24950061 PMC4078585

[39] Gilbert M, Chaitaweesub P, Parakamawongsa T, Premashthira S, Tiensin T, Kalpravidh W, et al Free-grazing ducks and highly pathogenic avian influenza, Thailand. Emerging infectious diseases. 2006;12(2):227 10.3201/eid1202.050640 16494747 PMC3373083

[40] Tiensin T, Nielen M, Songserm T, Kalpravidh W, Chaitaweesub P, Amonsin A, et al Geographic and temporal distribution of highly pathogenic avian influenza A virus (H5N1) in Thailand, 2004–2005: an overview. Avian diseases. 2007;51(s1):182–8. 10.1637/7635-042806R.1 17494551

[41] Stallknecht D, Kearney M, Shane S, Zwank P. Effects of pH, temperature, and salinity on persistence of avian influenza viruses in water. Avian diseases. 1990:412–8. 10.2307/1591429 2142421

[42] Cappelle J, Girard O, Fofana B, Gaidet N, Gilbert M. Ecological modeling of the spatial distribution of wild waterbirds to identify the main areas where avian influenza viruses are circulating in the Inner Niger Delta, Mali. EcoHealth. 2010;7(3):283–93. 10.1007/s10393-010-0347-5 20865438

[43] Pfeiffer DU, Minh PQ, Martin V, Epprecht M, Otte MJ. An analysis of the spatial and temporal patterns of highly pathogenic avian influenza occurrence in Vietnam using national surveillance data. The Veterinary Journal. 2007;174(2):302–9. 10.1016/j.tvjl.2007.05.010 17604193

