# Correction: Possible ramifications of climate variability on HPAI-H5N1 outbreak occurrence: Case study from the Menoufia, Egypt

**DOI:** 10.1371/journal.pone.0244360

**Published:** 2020-12-16

**Authors:** Yumna Elsobky, Gamal El Afandi, Ehsan Abdalla, Ahmed Byomi, Gopal Reddy

There are errors in the Author Contributions. The correct contributions are: Data curation, Methodology, Formal analysis, and Software: Yumna Elsobky and Gamal El Afandi. Writing original draft: Yumna Elsobky. Review and editing and Validation: Gamal El Afandi. Investigation and Supervision: Ehsan Abdalla, Gopal Reddy, Ahmed Byomi.
